# Increasing trends in central obesity among Chinese adults with normal body mass index, 1993–2009

**DOI:** 10.1186/1471-2458-13-327

**Published:** 2013-04-10

**Authors:** Tingting Du, Xingxing Sun, Ping Yin, Rui Huo, Chaochao Ni, Xuefeng Yu

**Affiliations:** 1 Department of Endocrinology, Tongji Hospital, Tongji Medical College of Huazhong University of Science and Technology, Wuhan, 430030, PR China; 2 Department of Anesthesiology, School of Stomatology, Fourth Military Medical University, Xi'an, 710032, PR China; 3 Department of Epidemiology and Biostatistics, School of Public Health, Tongji Medical College, Huazhong University of Science and Technology, Wuhan, 430030, PR China

**Keywords:** Body mass index, Waist circumference, Central obesity, General obesity, CHNS

## Abstract

**Background:**

Central obesity is thought to be more pathogenic than overall obesity and studies have shown that the association between waist circumference (WC) and mortality was strongest in those with a normal body mass index (BMI). The objective of our study was to determine secular trends in the prevalence of central obesity (WC ≥ 90 cm for men and ≥ 80 cm for women) among Chinese adults with normal BMI from 1993 to 2009 and to examine the impact of performance of combined BMI and WC on the prevalence of obesity in Chinese adults.

**Methods:**

We used data from the China Health and Nutrition Survey (CHNS) conducted from 1993 to 2009. From which we included a total of 52023 participants aged ≥ 18 years.

**Results:**

The age-standardized prevalence of central obesity among Chinese adults with BMI < 25 kg/m^2^ increased from 11.9% in 1993 to 21.1% in 2009 (P for linear trend <0.001). The upward trends were noted in both genders, all ages, rural/urban settings, and education groups (all P for linear trend <0.001), with greater increments in men, participants aged 18–64 years, and rural residents (P for interaction terms survey × sex, survey × age, and survey × rural/urban settings were 0.042, 0.003, and < 0.001, respectively). Trends in the prevalence of central obesity were similar when a more stringent BMI < 23 kg/m^2^ cut point (Asian cut point) was applied. Central obesity is associated with a higher risk of incident hypertension within normal BMI category. More than 65% individuals with obesity would be missed if solely BMI was measured.

**Conclusions:**

We observed an upward trend in the prevalence of central obesity among participants with normal BMI irrespective of sex, age, rural/urban settings, and education level. Central obesity is associated with a higher risk of incident hypertension within normal BMI category. Approximately two thirds of the individuals with obesity would be missed if WC was not measured. It is, therefore, urgent to emphasize the importance of WC as a measure to monitor the prevalence of obesity.

## Background

Despite the evidence that body mass index (BMI) is a key component of choice to provide a standardized definition of obesity for the purposes of national surveillance and international comparisons [1], the prevalence of obesity as defined by BMI should be interpreted cautiously as it is a poor indicator of body fat distribution [2]. Since waist circumference (WC) is strongly correlated with central fat localization [3], it is a sensitive measure of central-type obesity. Epidemiological studies have revealed that excess deposition of fat in the abdominal region seemed to be a risk factor for numerous adverse health outcomes independently of general adiposity reflected by BMI [4-6]. The prevalence of general obesity and central obesity as measured by BMI and WC, respectively, are well-documented and both of them have increased dramatically worldwide [7-14]. Interestingly, significant increase in the prevalence of central obesity seems to be more pronounced than that of general obesity at given periods in both China and other countries [8-10,13,15]. Hence, the diagnostic use of WC may to some degree have a particularly significant impact on the current epidemiological landscape of obesity. Studies have also illustrated that a larger increase in central obesity may not be related to the change in BMI [9]. Hence, it is very likely that a great number of subjects with normal BMI category may suffer from central obesity. Since the US National Institutes of Health recommended that WC be measured to screen for increased disease risk only among individuals in the overweight and obese categories of BMI [16], information on the prevalence of central obesity within normal category of BMI is scant [9,10], particularly in China. Relatively few health care professionals in China routinely measure WC, particularly in individuals with normal category of BMI. In addition, accumulating studies conducted in China indicated that central obesity was more important as a predictor of diabetes, hypertension, metabolic syndrome and cardiovascular disease than general obesity [17-19]. Furthermore, emerging evidence suggested that WC was also associated with higher risk of myocardial infarction or all-cause mortality within all categories of BMI [4,20]. Hence, our study aimed to describe the secular trends in the prevalence of central obesity among Chinese adults with normal BMI from 1993 to 2009, and to evaluate the impact on the prevalence of obesity if both BMI and WC were measured.

## Methods

### Study design

We used data from the China Health and Nutrition Survey (CHNS) for our analysis. The CHNS, an ongoing international collaborative project between the Carolina Population Center at the University of North Carolina at Chapel Hill and the National Institute of Nutrition and Food Safety at the Chinese Center for Disease Control and Prevention, is the only large-scale longitudinal, household-based survey in China [21]. It was designed to explore how the social and economic transformation of Chinese society is affecting the health and nutritional status of the Chinese population. The CHNS rounds were conducted in 1989, 1991, 1993, 1997, 2000, 2004, 2006, 2009 and 2011. For each round, a stratified multistage, random cluster process was employed to draw study sample from nine provinces (Liaoning, Heilongjiang, Jiangsu, Shandong, Henan, Hubei, Hunan, Guangxi and Guizhou), covering approximately 56% of China’s population, that vary significantly in terms of geography, economic development, and health status. Counties in the nine provinces were stratified by income (low, middle and high) and a weighted sampling scheme was used to select randomly four counties in each province. Full details of the CHNS have been described elsewhere [21]. Each participant provided a written informed consent and the study was approved by institutional review board from the University of North Carolina at Chapel Hill and the National Institute for Nutrition and Food Safety, China Center for Disease Control and Prevention.

### Study population

Since WC was initially collected in 1993, this study examined data from CHNS: 1993, 1997, 2000, 2004, 2006, and 2009. There were 64308 participants included in these six surveys. All participants were asked to complete a structured questionnaire which provided information on age, sex, degree of urbanization (urban vs. rural), educational attainment, a history of diabetes and so on. They also underwent detailed physical examinations that included weight, height, WC and blood pressure (BP). Participants were included in the present analysis if they were 18 years or older. Pregnant women or participants with missing information on age, WC, BMI, or BP, extreme or implausible WC (51 cm or 190 cm), BMI, or BP values were excluded from analysis. The remaining analytic sample sizes for the current study were 7274 for 1993, 8368 for 1997, 9369 for 2000, 8948 for 2004, 8786 for 2006, and 9278 for 2009.

### Measurements

Weight, height, WC and BP were measured following standardized protocols from the World Health Organization (WHO) [22,23]. Weight was measured with the participants wearing light clothing on a calibrated beam scale and height was measured without shoes using a portable stadiometer. BMI was calculated as weight (kilogram) divided by squared height (meter), rounded to the nearest tenth. WC was measured with an inelastic tape to the nearest 0.1 cm at a midpoint between the bottom of the rib cage and the top of the iliac crest at the end of exhalation. BP was measured by trained examiners using a mercury sphygmomanometer at three different consecutive times at 3–5 min intervals on one visit. The three readings were averaged as the BP values in our data analysis. All physical examinations were performed at the same location and followed the same protocol at each study visit.

### Definitions

According to WHO suggestions [22], normal weight is defined as BMI < 25 kg/m^2^, general obesity is defined as BMI ≥ 30 kg/m^2^. According to WHO expert consultation for Asians [24], normal weight is defined as BMI < 23 kg/m^2^. According to the criteria recommended by Working Group on Obesity in China [25], general obesity is defined as BMI ≥ 28 kg/m^2^. According to the International Diabetes Federation recommendations for Asians [26], central obesity is defined as WC ≥ 90 cm for men and ≥ 80 cm for women. According to the Seventh Joint National Committee on Prevention, Detection, Evaluation, and Treatment of High Blood Pressure guidelines [23], hypertension is defined as systolic BP ≥ 140 mmHg, diastolic BP ≥ 90 mmHg, and/or self-reported treatment of hypertension with antihypertensive medication in the last 2 weeks.

### Statistical analysis

All statistical analyses were conducted using SPSS software (version 16.0 for windows; SPSS, Chicago, IL, USA). Continuous variables were presented as means and standard deviations (SD). Categorical variables were expressed as numbers or percentages. One-way ANOVA was applied to compare differences in means across groups. A Chi-square test was performed to assess differences of proportions across groups. Analyses were stratified by sex, age group (18–44 years, 45–64 years, and of 65 years or older), degree of urbanization (urban vs. rural), and educational attainment (less than high school, high school, and university). The estimated prevalence was age-standardized to 2000 census of the Chinese adult population by the direct method. Trends in the prevalence of central obesity among participants with BMI < 25 kg/m^2^ from 1993 to 2009 were assessed by Cochran-Armitage trend testing. A sensitivity analysis was performed using a more stringent BMI < 23 kg/m^2^ cut point (Asian cut point) rather than the WHO BMI < 25 kg/ m^2^ cut point. To assess whether changes throughout the 17-year period differed by sex, logistic regression analysis was utilized to examine potential interaction effects between cohort and sex. Similar processes were repeated separately for age, rural/urban and education groups. Venn diagram was constructed as a visual display of central obesity based on WC and general obesity based on BMI. Of 7274 individuals participated in 1993, we excluded 1351 participants with hypertension and 440 participants who did not attend any follow-up visits. The final sample size for incidence analyses of hypertension was 5483 persons. Cox proportional-hazards models were used to predict the incident hypertension from WC within BMI categories. A two-tailed *P* value of < 0.05 was considered to be statistically significant.

## Results

The percentages of women remained relatively stable during the period covered by the surveys (P for trend > 0.05) (Table 1). The mean age increased successively from 1993 to 2009 (P < 0.01).

**Table 1:** Unadjusted demographic characteristics of Chinese adults aged ≥ 18 years: the CHNS 1993–2009

	**1993**	**1997**	**2000**	**2004**	**2006**	**2009**
	**(*n *= 7274)**	**(*n *= 8368)**	**(*n *= 9369)**	**(*n *= 8948)**	**(*n *= 8786)**	**(*n *= 9278)**
Women (%)	52.0	51.1	51.6	52.1	52.9	52.4
Age (y, Mean ± SD)	42.1 ± 15.6	43.5 ± 15.8	44.9 ± 15.5	48.0 ± 15.3	49.1 ± 15.1	50.6 ± 15.3

### Secular trends in the prevalence of central obesity among participants with BMI < 25 kg/m^2^

Detailed information on trends in the age-standardized prevalence of central obesity according to WHO criteria for Asians among subjects with BMI < 25 kg/m^2^ were shown for overall and by sex, age, degree of urbanization, and educational attainment in Table 2. The prevalence of central obesity doubled approximately from 11.9% in 1993 to 21.1% in 2009 (P for linear trend < 0.001). In stratified analysis, although the prevalence among women approximately tripled that among men in each survey (all P < 0.001), the change in the prevalence of central obesity was more striking among men than that among women, with the prevalence escalated from 4.4 to 10.4% among men and from 19.3 to 30.9% among women. The gender dependent changes were further confirmed by the significant interaction terms (survey × sex) (p = 0.042). For each survey, the age-specific prevalence of central obesity increased with age (all P for trend < 0.01). In addition, for each age group, the prevalence of central obesity increased linearly across the study periods (P < 0.001). However, the prevalence of central obesity increased more rapidly among participants 18–64 years of age than that among those aged ≥ 65 years (P = 0.003 for interaction terms survey × age). The prevalence of central obesity increased progressively over the six study periods in both rural and urban settings (both P for trend < 0.001). Notably, the change in the prevalence was particularly pronounced among participants residing in rural settings (P < 0.001 for interaction terms survey × rural/urban settings). For each education group, the prevalence of central obesity increased progressively over the 6 surveys (all P for trend <0.001). Although the prevalence was much higher among participants in the lowest education group (less than high school) than among those in the middle education group (high school) or the highest education group (university) (P < 0.001 for each survey), the increments in the prevalence of central obesity did not differ by education groups (P = 0.67 for interaction terms survey × education groups).

**Table 2:** Age-standardized prevalence of central obesity^* ^**among Chinese adults with BMI < 25 kg/m**^**2**^

	**1993**		**1997**		**2000**		**2004**		**2006**		**2009**		**P for trend**^**†**^	**P **^**‡**^
	**n**	**% (SE)**	**n**	**% (SE)**	**n**	**% (SE)**	**n**	**% (SE)**	**n**	**% (SE)**	**n**	**% (SE)**		
Total	5060	11.9 (0.4)	5346	12.2 (0.4)	5281	15.8 (0.4)	4722	18.2 (0.5)	4485	18.6 (0.5)	4597	21.1 (0.5)	< 0.001	
Men	2538	4.4 (0.4)	2695	4.6 (0.4)	2652	7.7 (0.4)	2274	10.1 (0.5)	2119	8.9 (0.5)	2160	10.4 (0.5)	< 0.001	0.042
Women	2522	19.3 (0.7)	2651	19.8 (0.7)	2629	23.8 (0.7)	2448	26.0 (0.8)	2366	27.4 (0.8)	2437	30.9 (0.8)	< 0.001	
Age (years)														
18-44	3228	7.7 (0.4)	3202	8.4 (0.4)	2941	11.0 (0.5)	2229	13.5 (0.6)	2054	13.6 (0.7)	1948	15.1 (0.7)	< 0.001	0.003
45-64	1351	17.7 (0.9)	1515	17.7 (0.8)	1665	22.3 (0.8)	1727	24.9 (0.9)	1629	25.5 (0.9)	1770	34.9 (0.9)	< 0.001	
65-118	481	23.1 (1.7)	629	22.2 (1.5)	675	29.8 (1.5)	766	30.2 (1.4)	802	32.0 (1.4)	879	35.3 (1.4)	< 0.001	
Region														
Urban	1407	15.7 (0.9)	1694	12.7 (0.7)	1640	16.3 (0.8)	1497	18.4 (0.8)	1432	19.8 (0.9)	1461	20.8 (0.9)	< 0.001	< 0.001
Rural	3653	9.6 (0.4)	3652	11.3 (0.5)	3641	14.7 (0.5)	3225	17.2 (0.6)	3053	17.1 (0.6)	3136	20.1 (0.6)	< 0.001	
Education														
Less than high school	4226	12.8 (0.5)	4347	13.0 (0.5)	4162	16.5 (0.5)	3690	19.0 (0.6)	3411	18.9 (0.6)	3441	22.4 (0.6)	< 0.001	0.67
High school	706	9.3 (1.0)	827	10.0 (0.9)	895	14.8 (1.0)	595	17.3 (1.3)	588	17.5 (1.3)	582	19.6 (1.4)	< 0.001	
University	128	10.5 (2.3)	172	11.1 (2.1)	224	12.2 (1.9)	437	14.5 (1.4)	486	17.0 (1.4)	574	16.4 (1.3)	< 0.001	

SE, standard error.

^*^ Central obesity is defined as waist circumference ≥ 90 cm for men and ≥ 80 cm for women.

^†^ Trends in the prevalence of central obesity from 1993 to 2009 were assessed by Cochran-Armitage trend testing.

^‡^ P for interaction terms (survey × sex, survey × age, survey × rural/urban settings, and survey × education group) were assessed by logistic regression analysis.

When a more stringent BMI < 23 kg/m^2^ cut point (Asian cut point) rather than the BMI < 25 kg/m^2^ cut point was applied, results were similar (Table 3), but with a relatively lower prevalence of central obesity. Furthermore, the increments in the prevalence of central obesity among participants with BMI < 23 kg/m^2^ during the period 1993–2009 did not differ by sex and age (P = 0.49 and 0.18 for interaction terms survey × sex and survey × age, respectively).

**Table 3:** Age-standardized prevalence of central obesity^* ^**among Chinese adults with BMI < 23 kg/m**^**2**^

	**1993**		**1997**		**2000**		**2004**		**2006**		**2009**		**P for trend**^**†**^	**P **^**‡**^
	**n**	**% (SE)**	**n**	**% (SE)**	**n**	**% (SE)**	**n**	**% (SE)**	**n**	**% (SE)**	**n**	**% (SE)**		
Total	5060	7.6 (0.4)	5346	7.6 (0.4)	5281	10.2 (0.4)	4722	12.1 (0.5)	4485	11.5 (0.5)	4597	13.5 (0.6)	< 0.001	
Men	2538	2.5 (0.3)	2695	2.2 (0.3)	2652	4.5 (0.4)	2274	5.8 (0.5)	2119	4.3 (0.5)	2160	4.9 (0.5)	< 0.001	0.49
Women	2522	13.0 (0.7)	2651	13.3 (0.7)	2629	16.1 (0.8)	2448	18.2 (0.9)	2366	18.1 (0.9)	2437	21.2 (0.9)	< 0.001	
Age (years)														
18-44	3228	4.9 (0.4)	3202	5.4 (0.4)	2941	7.6 (0.5)	2229	9.3 (0.6)	2054	8.9 (0.7)	1948	9.6 (0.7)	< 0.001	0.18
45-64	1351	11.1 (0.9)	1515	10.4 (0.8)	1665	13.0 (0.9)	1727	15.6 (1.0)	1629	14.6 (1.0)	1770	18.3 (1.0)	< 0.001	
65-118	481	15.7 (1.8)	629	14.6 (1.5)	675	19.3 (1.7)	766	21.1 (1.7)	802	20.8 (1.6)	879	26.0 (1.7)	< 0.001	
Region														
Urban	1407	10.5 (0.9)	1694	7.6 (0.7)	1640	10.8 (0.8)	1497	12.3 (0.9)	1432	12.3 (1.0)	1461	13.0 (1.0)	< 0.001	< 0.0001
Rural	3653	6.2 (0.4)	3652	7.2 (0.4)	3641	9.4 (0.5)	3225	11.5 (0.6)	3053	10.6 (0.6)	3136	12.9 (0.7)	< 0.001	
Education														
Less than high school	4226	8.3 (0.5)	4347	8.3 (0.4)	4162	10.4 (0.5)	3690	12.6 (0.6)	3411	11.7 (0.6)	3441	14.3 (0.7)	< 0.001	0.73
High school	706	6.2 (1.2)	827	5.6 (0.9)	895	10.3 (1.1)	595	12.1 (1.6)	588	10.9 (1.5)	582	12.1 (1.8)	< 0.001	
University	128	8.0 (2.5)	172	6.8 (2.0)	224	8.8 (2.0)	437	9.7 (1.5)	486	11.5 (1.5)	574	11.6 (1.4)	< 0.001	

SE, standard error.

^*^ Central obesity is defined as waist circumference ≥ 90 cm for men and ≥ 80 cm for women.

^†^ Trends in the prevalence of central obesity from 1993 to 2009 were assessed by Cochran-Armitage trend testing.

^‡^ P for interaction terms (survey × sex, survey × age, survey × rural/urban settings, and survey × education group) were assessed by logistic regression analysis.

### Overlap between obesity diagnosed by BMI and WC criteria

**Figure 1:**
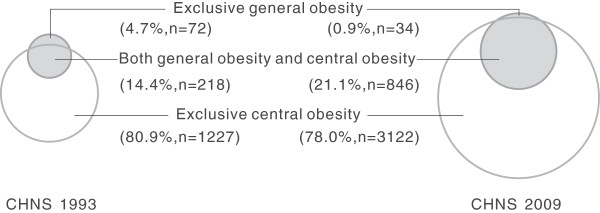
**Venn Diagrams for obesity based on either WC or BMI criteria.** WC criterion for obesity: waist circumference (WC) ≥ 90 cm for men and ≥ 80 cm for women. BMI criterion for obesity: body mass index (BMI) ≥ 28 kg/m^2^.

For each survey, among those identified to have general or central obesity based on a combination of BMI ≥ 28 kg/m^2^ and WC ≥ 90/80 cm, central obesity detected by WC occupied almost the whole percentage (≥ 95.3%) (1-the percentage of exclusive BMI ≥28 kg/m^2^); Most individuals who were identified with general obesity according to BMI criterion were also suffered from central obesity, as few participants (≤ 4.7%) were diagnosed exclusively with general obesity; Considerable participants (≥ 75.7%) were diagnosed exclusively with central obesity (Figure 1 and Table 4). Similar trends were noted in both genders for each survey. The prevalence of exclusively general obesity among men significantly exceeded that among women in each survey (all P < 0.05). Of note, the proportions of exclusive central obesity based on WC criterion surpassed 65% in each subgroup.

**Table 4:** Overlap^* ^**between BMI- and WC- based obesity among Chinese adults**

	**1993**		**1997**		**2000**		**2004**		**2006**		**2009**	
	**n**	**%**	**n**	**%**	**n**	**%**	**n**	**%**	**n**	**%**	**n**	**%**
Total												
BMI ≥ 28 kg/m^2^ or WC ≥ 90/80 cm	1517	…	2097	…	3021	…	3362	…	3469	…	4002	…
Exclusive BMI ≥ 28 kg/m^2^	72	4.7	64	3.1	86	2.8	103	3.1	105	3.0	34	0.9
Exclusive WC ≥ 90/80 cm	1227	80.9	1626	77.5	2296	76.0	2555	76.0	2627	75.7	3122	78.0
Both BMI ≥ 28 kg/m^2^and WC ≥ 90/80 cm	218	14.4	407	19.4	639	21.2	704	20.9	737	21.3	846	21.1
Men												
BMI ≥ 28 kg/m^2^ or WC ≥ 90/80 cm	387	…	648	…	992	…	1128	…	1146	…	1363	…
Exclusive BMI ≥ 28 kg/m^2^	47	12.1	49	7.5	49	4.9	66	5.9	70	6.1	28	2.1
Exclusive WC ≥ 90/80 cm	270	69.8	430	66.4	676	68.2	780	69.1	794	69.3	985	72.3
Both BMI ≥ 28 kg/m^2^and WC ≥ 90/80 cm	70	18.1	169	26.1	267	26.9	282	25.0	282	24.6	350	25.6
Women												
BMI ≥ 28 kg/m^2^ or WC ≥ 90/80 cm	1130	…	1449	…	2029	…	2234	…	2323	…	2639	…
Exclusive BMI ≥ 28 kg/m^2^	25	2.2	15	1.1	37	1.8	37	1.7	35	1.5	6	0.2
Exclusive WC ≥ 90/80 cm	957	84.7	1196	82.5	1620	79.8	1775	79.5	1833	78.9	2137	81.0
Both BMI ≥ 28 kg/m^2^ and WC ≥ 90/80 cm	148	13.0	238	16.4	372	18.4	422	18.8	455	19.6	496	18.8

^*^Proportions of obesity based on both WC ≥ 90/80 cm and BMI ≥ 28 kg/m^2^ (overlap between BMI- and WC- based obesity) is calculated as the number of obesity based on both WC ≥ 90/80 cm and BMI ≥ 28 kg/m^2^ divided by the number of obesity based on either WC ≥ 90/80 cm or BMI ≥ 28 kg/m^2^.

Likewise, proportions of exclusive general obesity and exclusive central obesity are calculated by the similar procedure.

Results were remarkably similar when a sensitivity analysis was conducted: using the BMI ≥ 30 kg/m ^2^ rather than the more restrictive Asian BMI ≥ 28 kg/m^2^ cut point to define general obesity, but with an even lower prevalence of exclusive general obesity (Additional file 1).

### HRs for high WC-related hypertension

In participants with BMI < 25 kg/m^2^, the hazard ratio (HR) for incident hypertension from central obesity compared with those with normal WC was 1.8 (95% CI, 1.6 to 2.0) (Table 5). After adjustment for confounding variables, this association was attenuated (1.4, 1.2 to 1.6 in model 1, and 1.3, 1.1 to 1.5 in in model 2) but still significant (P < 0.01). When a more restrictive BMI cut point (BMI < 23 kg/m^2^) was applied, results were similar.

**Table 5:** Hazard ratios for high WC-related incidence of hypertension within each BMI category

	**BMI categories**				**BMI categories**			
	**BMI < 25 kg/m**^**2**^		**BMI ≥ 25 kg/m**^**2**^		**BMI < 23 kg/m**^**2**^		**BMI ≥ 23 kg/m**^**2**^	
	**WC < 90/80 cm**	**WC ≥ 90/80 cm**	**WC < 90/80 cm**	**WC ≥ 90/80 cm**	**WC < 90/80 cm**	**WC ≥ 90/80 cm**	**WC < 90/80 cm**	**WC ≥ 90/80 cm**
Unadjusted model (HR, 95% CI)	1	1.8 (1.6-2.0)	1	1.1 (0.9-1.4)	1	1.6 (1.3-2.0)	1	1.5 (1.3-1.8)
Adjusted model 1^*^ (HR, 95% CI)	1	1.4 (1.2-1.6)	1	1.0 (0.7-1.4)	1	1.2 (1.0-1.6)	1	1.3 (1.1-1.6)
Adjusted model 2^†^ (HR, 95% CI)	1	1.3 (1.1-1.5)	1	1.0 (0.7-1.3)	1	1.2 (1.0-1.4)	1	1.2 (1.0-1.5)

^*^Adjusted model 1 was adjusted for sex, age, degree of urbanization, education, smoking status, alcohol consumption.

^†^ Adjusted model 2 was adjusted for all the variables in model 2 plus systolic blood pressure and diastolic blood pressure.

BMI, body mass index; WC, waist circumference, HR, hazard ratio, CI, confidence interval.

## Discussion

The present data provide evidence that the prevalence of central obesity among Chinese adults with BMI < 25 kg/m^2^ increased significantly from 1993 to 2009. The upward trend was noted irrespective of sex, age, rural/urban settings, and education groups. Notably, the prevalence of central obesity increased more rapidly among men than that among women. The increment was more prominent among participants aged 18–64 years than that among those aged ≥65 years. Participants residing in rural settings saw a more rapid increase in the prevalence compared with counterparts residing in urban settings. Central obesity is associated with a higher risk of incident hypertension within normal BMI category. Approximately two thirds of the individuals with obesity would be missed if screening by BMI alone.

As regards the prevalence of central obesity in subjects with BMI < 25 kg/m^2^, the findings from our present study are in line with the results obtained in the Pilot Study of the Fitness of Australians, where 6.5% of men and 22.0% of women with BMI < 25 kg/m^2^ are in a risk category based on WC [15]. In addition, two studies suggest an independent increase in the prevalence of central obesity over and above the prevalence of general obesity in adults [9,10]. Besides, a number of studies demonstrate a steeper rise in the prevalence of central obesity than the prevalence of general obesity although these studies do not indicate whether the central obesity has moved independently further than the general obesity [8,10,13]. Our finding that an increasing number of people with BMI < 25 kg/m^2^ are at risk for obesity due to excessive WC (≥ 90/80 cm) emphasizes the huge potential for preventing an unexpectedly large burden of obesity that remains to be realized in Chinese adults with BMI < 25 kg/m^2^. A recent study showed that among women, the association between WC and mortality was strongest in those with a normal BMI [20]. In addition, we found that approximately two thirds of individuals with obesity would be missed if WC is not taken into account for identification of obesity. An understanding of this issue is important for clinical reasons as weight management is a key strategy to the treatment of people with metabolic disturbance [27,28]. Therefore, obese cases with metabolic disturbance deserve high priority in risk factor modification. Evidence from studies show that achieving healthy weight through controlling weight adequately in obese patients could decrease blood pressure levels, improve lipid profile and insulin resistance [27]. The current study also shows that a higher WC is associated with a higher risk of incident hypertension within normal BMI category. It is of particular concern that most individuals in China on a visit to a doctor undergo assessment of body composition only by BMI for convenience. Given that weight reduction has important implications to reduce the burden of obesity-related disease, it is, therefore, necessary and crucial to make efforts to promote the measurement of WC in clinical practice. In addition, evidence from studies show that a substantial number of subjects with metabolic disturbance do not always conform to general obesity assessed by BMI. The degree of adiposity associated with a given level of BMI varies by racial and ethnic groups. Relative to blacks, a BMI of 20–25 kg/m^2^, which would be considered lean within blacks, corresponds to an elevated body fat content in Asian populations as they tend to have higher body fat percentages at this BMI level and possible less favorable health [29-31]. Indeed, BMI may simply reflect increased muscle mass in athletes, which does not brought them less favorable health as lean body mass is inversely related to all-cause mortality [32]. Hence, measuring WC could provide additional meaningful information beyond that provided by BMI for accurately predicting obesity-associated complications and an opportunity for proper intervention.

The finding from the current study that central obesity is observed more commonly in females than in males is similar to the results from other studies [8,11]. For instance, the prevalence of central obesity was 16.1% in men and 37.6% in women in the study of InterASIA [11]. One possible explanation for our tripling central obesity prevalence in women might be attributable to the fact that we adopted a threshold of 80 cm to diagnose central obesity in women and the mean WC of women is close to this cut-off point (data not shown), it is likely that women are more prone to exceed this threshold to be defined as central obesity when their WC increases. However, the root causes that lead to differential prevalence of central obesity in men and women warrants further study.

Explanations for the higher prevalence of exclusive general obesity in men than that in women might be due to the differences in body composition between men and women, with men tending to have a greater skeletal muscle than women and women tending to have a greater percentage of body fat than men [33,34], which further supported the notion that the performance of BMI in distinguishing fat mass from lean mass is limited.

Similar to the study of ENRICA [8], the frequency of central obesity increases with age in our study. Evidence from a study demonstrates that aging is associated with an increased accumulation of central fat [35]. Given a greater increment in the prevalence of central obesity noted among participants aged 18–64 years in our study, it is likely that older populations would bear a huge burden of central obesity when the relatively younger participants getting old. China has experienced extremely rapid increases in economic development, national wealth and lifespan over the last 20 years. Old populations within this transitional country, who have traditionally suffered from facing famine and food shortages, may eventually come to experience the highest risk of central obesity, indicating that urgent implementation of obesity prevention programs in old adults should be prioritized.

Our finding that rural residents saw a more rapid increase in the prevalence of central obesity compared with urban counterparts is also a characteristic observed in other population-based studies in China [13,36]. Given its large population living in rural setting, China may bear a high obesity related burden. Finally, our finding that a higher prevalence of central obesity observed in participants in the lowest education group is in keeping with the results from the previous studies [9,37]. Our results, together with those of previous studies [10,11,13], provide evidence that the burden of obesity also affects the lower social classes in China as educational attainment reflects socioeconomic status to some extent.

Our study has several limitations. First, the sample is partial nationally representative as only nine of China’s 31 provinces are included, and therefore, extrapolating results to the whole of China should be interpreted cautiously. Second, other social and environmental variables such as dietary habits and sedentary lifestyle, which would have impact on obesity, were not considered. Third, estimates across subgroups should also be interpreted with caution because of limited sample size. However, our study has several strengths. Firstly, our study maintains a large sample size and includes individuals from urban and rural settings in China, which allows for exploring the prevalence of obesity over a range of demographic groups. Secondly, all study measurements are made by trained staff following a standard protocol. A vigorous quality assurance program and the same strict methodology are used to ensure the quality of the data collection over the entire study period.

## Conclusions

The prevalence of central obesity increased significantly among Chinese adults with normal BMI from 1993 to 2009. The upward trends were noted in genders, all ages, rural/urban settings, and education groups, with greater increases in men, younger participants, and rural residents. Central obesity is an independent risk for incident hypertension in individuals with normal BMI. About 2 in 3 Chinese obese adults would be missed if solely BMI was adopted to define obesity. Results from our present study emphasize the importance of WC for monitoring the prevalence of obesity. Given the stronger association between WC and metabolic risk, measuring both BMI and WC is helpful in more accurately predicting obesity-associated health burden.

## Abbreviations

BMI: Body mass index; WC: Waist circumference; CHNS: China Health and Nutrition Survey; BP: Blood pressure; WHO: World Health Organization; SD: Standard deviation.

## Competing interests

The authors declare that they have no competing interests.

## Authors’ contributions

XFY, TTD conceived the study. XXS, TTD and PY completed all statistical analyses. XXS and TTD drafted the manuscript. XFY, RH, and CCN contributed to the discussion. XFY revised the manuscript. All authors have read and approved the final manuscript.

## Pre-publication history

The pre-publication history for this paper can be accessed here:

http://www.biomedcentral.com/1471-2458/13/327/prepub

## Supplementary Material

Additional file 1 Overlap^*^ between BMI- and WC- based obesity among Chinese adults. Click here for file
