# Supplementary material for: Increasing trends in central obesity among Chinese adults with normal body mass index, 1993–2009
Source: BMC Public Health. 2013 Apr 10;13:327. doi: 10.1186/1471-2458-13-327 (PMC3626835; doi:10.1186/1471-2458-13-327)
Supplement: Additional file 1 — Overlap* between BMI- and WC- based obesity among Chinese adults. [file 1471-2458-13-327-S1.docx]

Additional file 1

Overlap^＊^ between BMI- and WC- based obesity among Chinese adults

|  | 1993 | | 1997 | | 2000 | | 2004 | | 2006 | | 2009 | |
| --- | --- | --- | --- | --- | --- | --- | --- | --- | --- | --- | --- | --- |
|  | n | % | n | % | n | % | n | % | n | % | n | % |
| Total |  |  |  |  |  |  |  |  |  |  |  |  |
| BMI ≥ 30 kg/m^2^ or WC ≥ 90/80 cm | 1498 | … | 2080 | … | 2986 | … | 3326 | … | 3428 | … | 3977 | … |
| Exclusive BMI ≥ 30 kg/m^2^ | 53 | 3.5 | 47 | 2.3 | 51 | 1.7 | 67 | 2.0 | 64 | 1.89 | 9 | 0.2 |
| Exclusive WC ≥ 90/80 cm | 1363 | 91.0 | 1876 | 90.2 | 2667 | 89.3 | 2932 | 88.2 | 3016 | 88.0 | 3600 | 90.5 |
| Both BMI ≥30 kg/m^2^and WC ≥ 90/80 cm | 82 | 5.5 | 157 | 7.5 | 268 | 9.0 | 327 | 9.8 | 348 | 10.2 | 368 | 9.3 |
| Men |  |  |  |  |  |  |  |  |  |  |  |  |
| BMI ≥ 30 kg/m^2^or WC ≥ 90/80 cm | 375 | … | 634 | … | 970 | … | 1101 | … | 1116 | … | 1341 | … |
| Exclusive BMI ≥ 30 kg/m^2^ | 35 | 9.3 | 35 | 5.5 | 27 | 2.8 | 39 | 3.5 | 40 | 3.6 | 6 | 0.5 |
| Exclusive WC ≥ 90/80 cm | 317 | 84.5 | 544 | 85.8 | 838 | 86.4 | 944 | 85.7 | 939 | 84.1 | 1176 | 87.7 |
| Both BMI ≥ 30 kg/m^2^and WC ≥ 90/80 cm | 23 | 6.2 | 55 | 8.7 | 105 | 10.8 | 118 | 10.7 | 137 | 12.3 | 159 | 11.9 |
| Women |  |  |  |  |  |  |  |  |  |  |  |  |
| BMI ≥ 30 kg/m^2^or WC ≥ 90/80 cm | 1123 | … | 1446 | … | 2016 | … | 2225 | … | 2312 | … | 2636 | … |
| Exclusive BMI ≥30 kg/m^2^ | 18 | 1.6 | 12 | 0.8 | 24 | 1.2 | 28 | 1.2 | 24 | 1.1 | 3 | 0.1 |
| Exclusive WC ≥ 90/80 cm | 1046 | 93.1 | 1332 | 92.1 | 1829 | 90.7 | 1988 | 89.4 | 2077 | 89.8 | 2424 | 92.0 |
| Both BMI ≥30 kg/m^2^ and WC ≥ 90/80 cm | 59 | 5.3 | 102 | 7.1 | 163 | 8.1 | 209 | 9.4 | 211 | 9.1 | 209 | 7.9 |

- Proportions of obesity based on both WC ≥ 90/80 cm and BMI ≥ 30 kg/m^2^ (overlap between BMI- and WC- based obesity ) is calculated as the number of obesity based on both WC ≥ 90/80 cm and BMI ≥ 30 kg/m^2^ divided by the number of obesity based on either WC ≥ 90/80 cm or BMI ≥ 30 kg/m^2^.

Likewise, proportions of exclusive general obesity and exclusive central obesity are calculated by the similar procedure.
